# Nitrogen-Dependent Regulation of De Novo Cytokinin Biosynthesis in Rice: The Role of Glutamine Metabolism as an Additional Signal

**DOI:** 10.1093/pcp/pct127

**Published:** 2013-10-10

**Authors:** Tomoe Kamada-Nobusada, Nobue Makita, Mikiko Kojima, Hitoshi Sakakibara

**Affiliations:** RIKEN Center for Sustainable Resource Science, Tsurumi, Yokohama, 230-0045 Japan

**Keywords:** Ammonium, *Arabidopsis thaliana*, Cytokinin, Glutamine, Nitrate, *Oryza sativa*

## Abstract

Cytokinin activity in plants is closely related to nitrogen availability, and an Arabidopsis gene for adenosine phosphate-isopentenyltransferase (IPT), *IPT3*, is regulated by inorganic nitrogen sources in a nitrate-specific manner. In this study, we have identified another regulatory system of cytokinin de novo biosynthesis in response to nitrogen status. In rice, *OsIPT4*, *OsIPT5*, *OsIPT7* and *OsIPT8* were up-regulated in response to exogenously applied nitrate and ammonium, with accompanying accumulation of cytokinins. Pre-treatment of roots with l-methionine sulfoximine, a potent inhibitor of glutamine synthetase, abolished the nitrate- and ammonium-dependent induction of *OsIPT4* and *OsIPT5*, while glutamine application induced their expression. Thus, neither nitrate nor ammonium, but glutamine or a related metabolite, is essential for the induction of these *IPT* genes in rice. On the other hand, glutamine-dependent induction of *IPT3* occurs in Arabidopsis, at least to some extent. In transgenic lines repressing the expression of *OsIPT4*, which is the dominant IPT in rice roots, the nitrogen-dependent increase of cytokinin in the xylem sap was significantly reduced, and seedling shoot growth was retarded despite sufficient nitrogen. We conclude that plants possess multiple regulation systems for nitrogen-dependent cytokinin biosynthesis to modulate growth in response to nitrogen availability.

The nucleotide sequence reported in this paper has been submitted to DDBJ under accession number AB853903 (OsIPT8).

## Introduction

Nitrogen is an essential but often limiting nutrient for plant growth and development. To regulate growth under limited nitrogen supply, plants sense the internal and external nitrogen status and coordinate various metabolic processes and developmental programs. To enable such an elaborate response, a wide range of genes are regulated in response to the nitrogen status and to intracellular, intercellular and interorgan signals.

Inorganic nitrogen sources differ depending on soil conditions: nitrate is the major form in most natural soils, whereas ammonium may dominate under reducing conditions, such as in paddy fields. In terms of nitrogen source-dependent gene regulation, two response modes were characterized in previous studies: the nitrate-specific response and the nitrogen-non-specific response (Sakakibara 2003b, Sakakibara et al. 2006, Castaings et al. 2011). As for the former, transcriptome analysis and other studies have identified a wide range of nitrate-responsive genes including nitrate-assimilatory and related genes (Sakakibara et al. 1996, Matsumura et al. 1997, Redinbaugh and Campbell 1998, Wang et al. 2000, Wang et al. 2003, Scheible et al. 2004, Sakakibara et al. 2006, Okamoto et al. 2009). This response is rapid and does not require de novo protein synthesis since it is cycloheximide insensitive (Gowri et al. 1992, Sakakibara et al. 1997, Sakakibara 2003a). Key transcription factors and *cis*-element sequences for the nitrate-specific response recently have been described (Castaings et al. 2009, Konishi and Yanagisawa 2010, Konishi and Yanagisawa 2011, Konishi and Yanagisawa 2013, Sawaki et al. 2013). On the other hand, the nitrogen-non-specific response is observed following exogenous application of nitrate, ammonium and various amino acids, and a wide spectrum of genes have been reported to be up- or down-regulated during the response (Hirose et al. 1997, Sivasankar et al. 1997, Stitt 1999, Stitt et al. 2002, Sonoda et al. 2003, Tabuchi et al. 2007, Konishi and Yanagisawa 2010, Kiba et al. 2012).

Recent studies indicated a linkage between the nitrogen signaling network and phytohormones including cytokinin, ABA and auxin (Kiba et al. 2011, Krouk et al. 2011, Ruffel et al. 2011). Cytokinin metabolism and signaling are closely related to nitrogen availability (Sakakibara et al. 1998, Taniguchi et al. 1998, Takei et al. 2001, Miyawaki et al. 2004, Takei et al. 2004, Ruffel et al. 2011). Cytokinin regulates a variety of processes in plant growth and development, such as shoot and root growth, apical dominance, leaf longevity, sink capacity and stress responses (Werner et al. 2003, Ashikari et al. 2005, Kim et al. 2006, Tanaka et al. 2006, Kitazawa et al. 2008, Bartrina et al. 2011, Kondo et al. 2011, F. Qin et al. 2011a; H. Qin et al. 2011b). *trans*-Zeatin (tZ), *cis*-zeatin (cZ) and *N*^6^-(Δ^2^-isopentenyl)adenine (iP) are common natural cytokinins in plants (Mok and Mok 2001, Sakakibara 2006). The concentrations of tZ, iP and their conjugates (tZ-type cytokinins and iP-type cytokinins, respectively) correlate with the supplied amounts of nitrogen (Samuelson and Larsson 1993, Kiba et al. 2011); nitrogen supplements elevate the cytokinin levels in *Zea mays* (maize) (Takei et al. 2001), *Hordeum vulgare* (barley) (Samuelson and Larsson 1993), *Arabidopsis thaliana* (Arabidopsis) (Takei et al. 2004), *Triticum aestivum* (wheat) (Garnica et al. 2010) and *Urtica dioica* (stinging nettle) (Wagner and Beck 1993). According to current understanding, cytokinins are synthesized in various parts of the plant body and act locally and at a distance. They may be systemically transported via the vasculature to coordinate shoot and root development (Matsumoto-Kitano et al. 2008, Kudo et al. 2010, Bishopp et al. 2011). This suggests that cytokinins could function as long-range signals communicating the rhizospheric nitrogen status.

The initial step of the de novo biosynthesis of iP- and tZ-type cytokinins is catalyzed by adenosine phosphate-isopentenyltransferase (IPT), producing the nucleotide precursors (Sakakibara 2006). In Arabidopsis, *IPT3*, which is expressed in phloem tissue, is up-regulated by exogenous nitrate (Miyawaki et al. 2004, Takei et al. 2004). The up-regulation is nitrate specific, and a loss-of-function mutation severely diminished the nitrate-dependent accumulation of cytokinin. This indicated that *IPT* is a key regulator of the nitrate-responsive modulation of cytokinin activity (Takei et al. 2004).

The nitrate-specific up-regulation of Arabidopsis *IPT3* is not the only mode of nitrogen-dependent regulation of cytokinin levels. For instance, the expression of the cytokinin-responsive maize response regulator genes, *ZmRR1* and *ZmRR2*, apparently is induced by exogenous nitrate as well as ammonium (Sakakibara et al. 1998, Sakakibara et al. 1999). In barley, pre-treatment with inhibitors of nitrate reductase (NR) and glutamine synthetase (GS) diminished the nitrate-dependent accumulation of cytokinin (Samuelson and Larsson 1993).

Paddy rice (*Oryza sativa*) utilizes ammonium as its major inorganic nitrogen source. Eight *IPT* genes (*OsIPT1–OsIPT8*) have been identified; their different organ specificities (Sakamoto et al. 2006) suggest functional differentiation. The dominant cytokinin species in rice are different from those in Arabidopsis: cZ and its conjugates (cZ-type cytokinins) are more abundant than tZ-type and iP-type cytokinins, although cZ activity is generally weak and equivalent to that of tZ only in specific contexts, such as the inhibition of seminal root elongation (Kojima et al. 2009, Choi et al. 2012, Kudo et al. 2012, Kudo et al. 2013). Therefore, insights into the regulation of cytokinin metabolism in response to nitrogen nutrition in rice may foster a deeper general understanding of the nitrogen-dependent regulation of cytokinin activity. In this study, we identified nitrogen-responsive *IPT* genes in rice and characterized their regulation. We found that a key signal for gene induction is glutamine or a related metabolite. Our results reveal mechanistic differences and commonalities in the nitrogen-dependent regulation of de novo cytokinin biosynthesis between rice and Arabidopsis.

## Results

### Changes in cytokinin concentrations in response to inorganic nitrogen sources

**Fig. 1:**
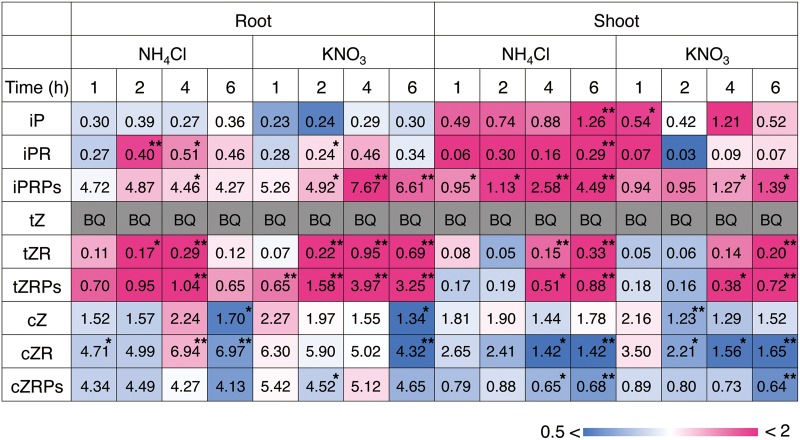
Accumulation pattern of cytokinins in rice roots and shoots in response to nitrate and ammonium. Rice seedlings were hydroponically grown in tap water for 11 d after sowing and transferred to nitrogen-free culture medium for 3 d. Then, the roots were dipped into culture media containing 1 mM NH_4_Cl, 1 mM KNO_3_ or 1 mM KCl. After the time indicated, roots and shoots were separately harvested in triplicate, and the cytokinin contents were quantified. The value in each block indicates the concentration (mean values) as pmol g^−1^ FW. The relative accumulation level of each compound compared with the accumulation in the KCl treatment is color-coded. BQ, below the quantification limit. *P < 0.05; **P < 0.01 (Student’s *t*-test, comparison with the KCl treatment). The complete data set is presented in Supplementary Tables S1 and S2.

To examine whether nitrogen nutrition affects cytokinin metabolism in rice, cytokinins and their conjugates were quantified in rice seedlings after supplement of nitrate or ammonium. In roots and shoots, iP riboside 5′-phosphates (iPRPs) and tZ riboside 5′-phosphates (tZRPs), early products of de novo cytokinin synthesis, accumulated following exposure to both nitrogen sources (Fig. 1; Supplementary Tables S1, S2). Similar patterns were observed in the nucleosides and active-form nucleobases, iP riboside (iPR), iP and tZ riboside (tZR), whereas tZ was below the quantification limit at all times. The accumulation of transcripts of cytokinin-responsive type-A response regulator genes was also increased in roots (Supplementary Fig. S1). On the other hand, there was no apparent increase in cZ, cZ riboside (cZR) and cZR 5′-phosphates (cZRPs). These results suggested that exposure to inorganic nitrogen sources activated the de novo synthesis of tZ- and iP-type cytokinins in rice.

### Identification of nitrogen-responsive *OsIPT* genes

**Fig. 2:**
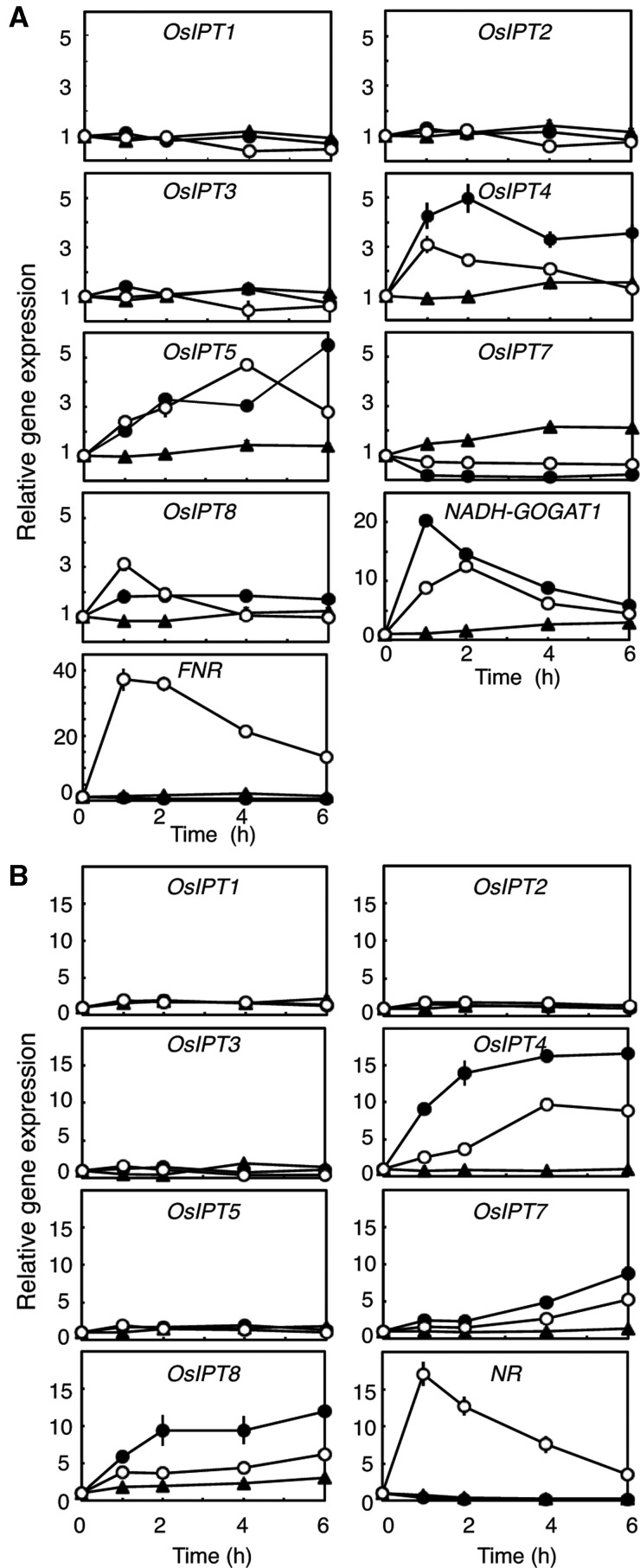
Changes in the accumulation of *OsIPT* transcripts in response to nitrogen sources in roots (A) and shoots (B). Rice seedlings were hydroponically grown and treated with 1 mM NH_4_Cl (filled circles), 1 mM KNO_3_ (open circles) or 1 mM KCl (filled triangles) in the same manner as in Fig. 1, and roots and shoots were harvested at the indicated times. Total RNA prepared from the samples was subjected to qPCR. The amounts of transcripts were normalized to the value at 0 min. qPCR was performed in triplicate, and mean values with the SD are shown.

Since nucleotide precursors of cytokinins were increased by the nitrogen supplements, we examined the accumulation levels of *OsIPT* transcripts in roots and shoots (Fig. 2). Since *OsIPT6* seems to be non-functional in the Nipponbare cultivar (Sakamoto et al. 2006), we excluded it from analysis. To discriminate between nitrate-specific and nitrogen-non-specific responses, genes for non-photosynthetic-type ferredoxin-NADP^+^ oxidoreductase (FNR) (Aoki and Ida 1994) and NADH-dependent glutamate synthase 1 (NADH-GOGAT1) (Hirose et al. 1997) were used as indicators for the two response types, respectively, in roots (Fig. 2A). The *NR* gene (Hamat et al. 1989) served as an indicator for nitrate-specific responses in shoots (Fig. 2B). In roots, the accumulation of *OsIPT4* and *OsIPT5* transcripts was clearly increased by both ammonium and nitrate (Fig. 2A). Ammonium had a more potent effect on the induction of *OsIPT4* expression than nitrate. On the other hand, the level of the *OsIPT7* transcript was decreased by both treatments. In shoots, the accumulation levels of *OsIPT4*, *OsIPT7* and *OsIPT8* transcripts were increased by both nitrogen sources, with ammonium having a stronger effect than nitrate (Fig. 2B). The expression patterns of the indicator genes did not suggest any nitrate effects caused by ammonium-containing media.

**Fig. 3:**
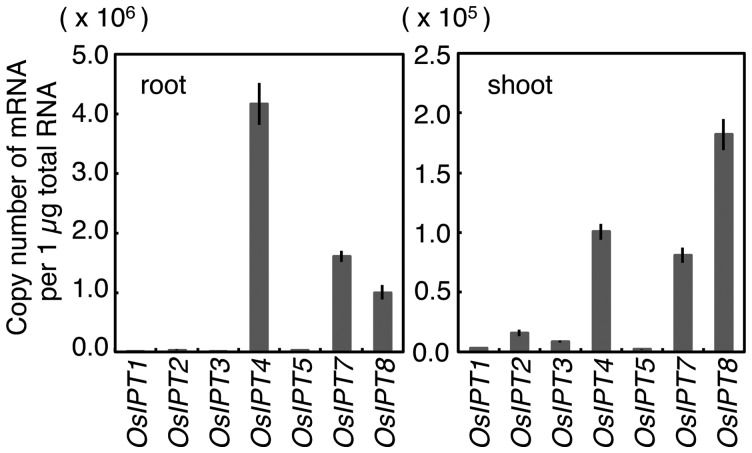
Accumulation of *OsIPT* transcripts in roots (left) and shoots (right) of rice seedlings hydroponically grown for 2 weeks in tap water. Total RNAs prepared from each organ were subjected to qPCR. The accumulations of transcripts are indicated as amounts per total RNA. qPCR was performed in triplicate, and mean values with the SD are shown.

When we analyzed the steady-state accumulation levels of *OsIPT* genes in rice seedlings before nitrogen application, we found *OsIPT4*, *OsIPT7* and *OsIPT8* transcripts abundantly accumulated in roots and shoots (Fig. 3). The *OsIPT4* transcript accumulated mostly in roots, while the *OsIPT8* transcript was most abundant in shoots. Accumulation levels of the *OsIPT5* transcript were much lower than those of other nitrogen-responsive *IPT* genes. These results suggested that the expression of *OsIPT4* on the one hand, and of *OsIPT4*, *OsIPT7* and *OsIPT8* on the other affected nitrogen-dependent cytokinin synthesis in roots and shoots, respectively.

### Spatial expression patterns of nitrogen-inducible *OsIPT* genes

**Fig. 4:**
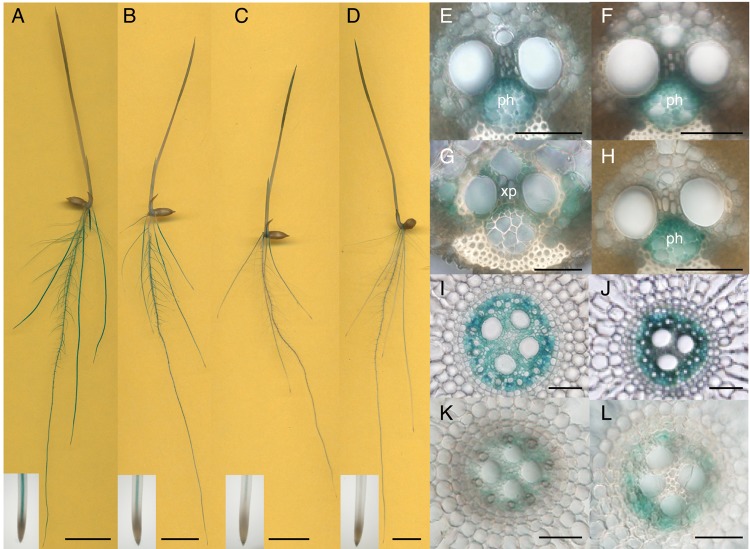
Distribution of GUS activity under the control of the 5′ upstream region of the *OsIPT4* (A, E and I), *OsIPT5* (B, F and J), *OsIPT7* (C, G and K) and *OsIPT8* (D, H and L) genes. (A–D) Whole transformant seedlings. Close-ups of the root apex are shown in the insets at the bottom. (E–L) Cross-sections of vascular bundles in mature leaf blades (E–H) and seedling roots (I–L). ph, phloem; xp, xylem parenchyma. Scale bars, 1 cm (A–D) and 50 µm (E–L).

To reveal the tissue specificities of the nitrogen-dependent cytokinin biosynthesis genes, DNA fragments containing about 3 kb of the upstream sequences including the N-terminal regions of *OsIPT4*, *OsIPT5*, *OsIPT7* and *OsIPT8* were fused with the *β-glucuronidase* (*GUS*) coding sequence, and the constructs (designated *OsIPT4pro:GUS*, *OsIPT5pro:GUS*, *OsIPT7pro:GUS* and *OsIPT8pro:GUS*) were transformed into rice. We detected similar GUS staining patterns with 10–13 independent T_1_ transgenic lines of each construct, and T_3_ representative lines were further analyzed in detail (Fig. 4). In these transgenic lines, GUS activity was detected in roots, and less so in shoots (Fig. 4A–D). This finding was consistent with the results in Fig. 3. In roots, GUS staining patterns were essentially the same for all *IPT* promoters: staining was observed in vascular bundles of seminal and crown roots (Fig. 4I–L) but not in the apical meristems (Fig. 4A–D, insets). The staining patterns in the transformants did not change with different nitrogen sources (data not shown).

In mature leaf blades of the transformants, GUS activity was also detected in the vascular bundles, but showed tissue specificity. In *OsIPT4pro:GUS*, *OsIPT5pro:GUS* and *OsIPT8pro:GUS* transformants, GUS activity was detected in the phloem, while in *OsIPT7pro:GUS* transformants it was found in xylem parenchyma cells (Fig. 4E–H).

### Intracellular localization of rice IPTs

In Arabidopsis, nitrogen-responsive IPT3 is localized in plastids (Kasahara et al. 2004). We examined the intracellular localization of OsIPTs in rice. Prior to this analysis, we conducted a 5′-rapid amplification of cDNA ends (RACE) analysis to predict the translation start site of OsIPT8 because of the discrepancy in the predicted lengths of the reading frame between Sakamoto et al. (2006) and a public database (Rice Genome Annotation Project; http://rice.plantbiology.msu.edu/). In our analysis, the first ATG codon of amplified cDNA appeared 45 bp upstream of that of the public database and 126 bp upstream of that reported by Sakamoto et al. (2006) (Supplementary Fig. S2).

**Fig. 5:**
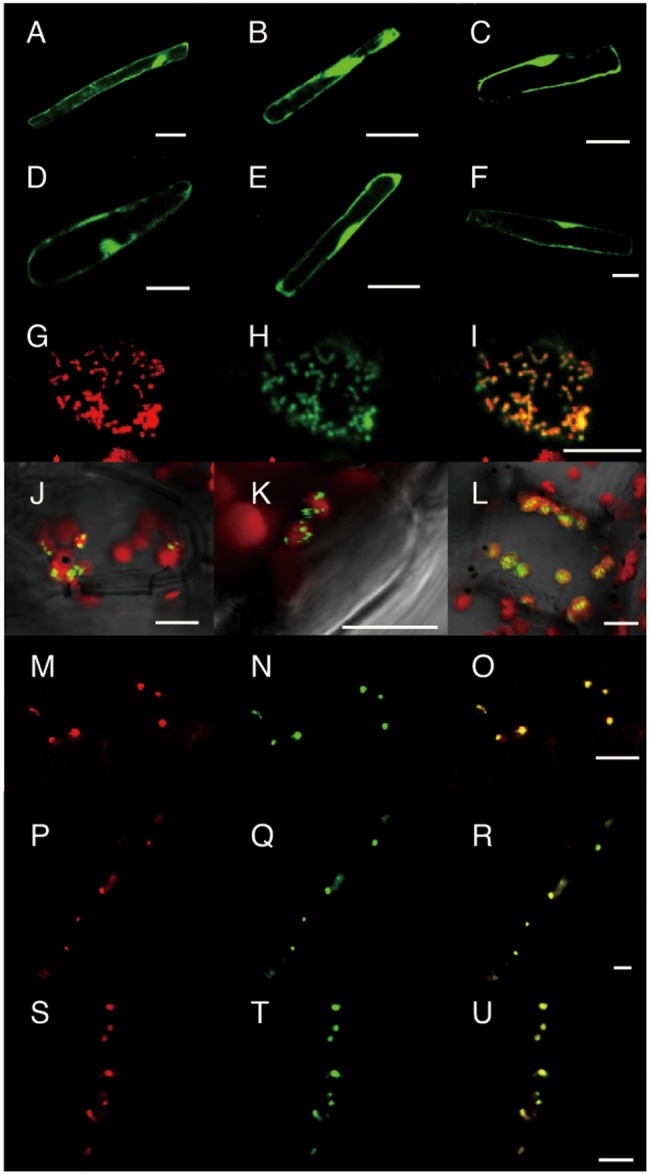
Subcellular localization of GFP-tagged OsIPT proteins in Arabidopsis observed by confocal laser-scanning microscopy. The translational fusion genes *OsIPT1-GFP* (A), *OsIPT2-GFP* (B), *OsIPT3-GFP* (C), *GFP-OsIPT1* (D), *GFP-OsIPT2* (E) and *GFP-OsIPT3* (F) were transiently expressed in root epidermal cells by particle bombardment. The fusion genes *pGGPS6-DsRed2* (G), a control for mitochondrial localization, and *OsIPT7-GFP* (H) were co-introduced into a leaf mesophyll cell. (I) Merged image of (G) and (H). The fusion genes *OsIPT4-GFP* (J), *OsIPT5-GFP* (K) and *OsIPT8-GFP* (L) were introduced into leaf mesophyll cells and superimposed on Chl autofluorescence (red). The fusion gene *AtFSD3-DsRed2* (M, P and S), a control for nucleoid localization, was co-introduced into root epidermal cells with either *OsIPT4-GFP* (N), *OsIPT5-GFP* (Q) or *OsIPT8-GFP* (T). (O, R and U) Merged images of (M) and (N), (P) and (Q), and (S) and (T), respectively. Scale bars, 20 µm (A–F) and 10 µm (G–U).

To examine the intracellular localization of OsIPT proteins, translational fusions were made with green fluorescent protein (GFP) at the C-termini (designated as OsIPT1–GFP to OsIPT8–GFP) under the control of the *Cauliflower mosaic virus* (CaMV) 35S promoter. The constructs were introduced into Arabidopsis cells by particle bombardment (Fig. 5). Since rice tissues are mechanically rigid and it was difficult to observe subcellular localizations, we used Arabidopsis as a heterologous system. GFP fluorescence of OsIPT4–GFP, OsIPT5–GFP and OsIPT8–GFP appeared as small dots on plastids (Fig. 5J–L). When we co-introduced DsRed2-fused pAtFSD3, a plastid nucleoid-associated protein (Myouga et al. 2008), the fluorescence signals fully overlapped (Fig. 5M–U). The GFP fluorescence of OsIPT7–GFP co-localized with DsRed2-tagged Arabidopsis geranylgeranyl diphosphate synthase 6 (GGPS6) (Okada et al. 2000), a control marker for mitochondria (Fig. 5G–I). These results strongly suggested that nitrogen-inducible OsIPT4, OsIPT5 and OsIPT8 are localized in plastids, whereas OsIPT7 is localized in mitochondria. On the other hand, the GFP fluorescence from OsIPT1–GFP, OsIPT2–GFP and OsIPT3–GFP and their translational fusions at the N-termini (GFP–OsIPT1, GFP–OsIPT2 and GFP–OsIPT3) were all observed in the cytoplasm (Fig. 5A–F).

### Metabolic signals for nitrogen-dependent *OsIPT* expression

**Fig. 6:**
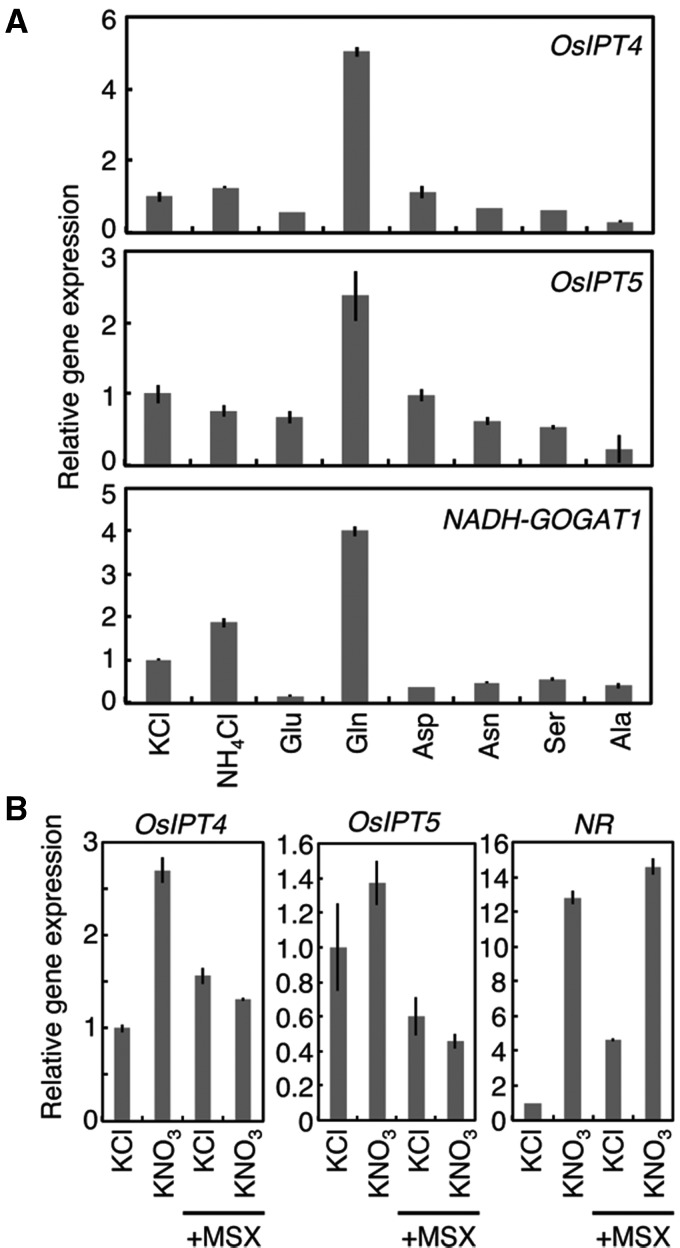
Effects of methionine sulfoximine (MSX) pre-treatment on the induction of *OsIPT* transcript accumulation by nitrogen compounds. Rice seedlings were hydroponically grown in tap water for 11 d after sowing and then on nitrogen-free culture medium for 3 d. The seedlings were pre-treated with 1 mM MSX for 2 h and then incubated with (A), 1 mM KCl, 1 mM NH_4_Cl or 50 mM of the indicated amino acids for 3 h; or (B), 10 mM KCl or 10 mM KNO_3_ for 3 h. In (B), samples without MSX pre-treatment were also prepared. Total RNA prepared from the roots was subjected to qPCR. The amounts of transcripts were normalized with respect to the value in the KCl treatment. qPCR was performed in triplicate, and mean values with the SD are shown.

To obtain information about the regulation of nitrogen-dependent *OsIPT* expression, we examined the effects of l-methionine sulfoximine (MSX), an inhibitor of GS (Pace and Mcdermott 1952). Roots of rice seedlings grown for 2 weeks without nitrogen were pre-treated with MSX before ammonium or amino acids were applied. Pre-treatment with MSX completely inhibited the ammonium-induced accumulation of *OsIPT4* and *OsIPT5* transcripts (Fig. 6A), indicating that ammonium itself is not a direct inducing signal. On the other hand, application of glutamine induced expression of *OsIPT* genes as well as that of *NADH-GOGAT1*, a glutamine-responsive gene (Hirose et al. 1997). Other amino acids had no comparable effects. Application of glutamine increased the cytokinin concentration in roots (Supplementary Fig. S3). These results suggested that glutamine or a related metabolite regulates the cytokinin concentration via nitrogen-dependent *OsIPT4* and *OsIPT5* expression.

In Arabidopsis, the nitrogen-responsive *AtIPT3* is induced in a nitrate-specific manner (Takei et al. 2004). To see whether the nitrate-specific response is conserved in *OsIPT* gene induction in rice, we examined the effects of nitrate on the expression of *OsIPT4*, *OsIPT5* and *NR* after MSX pre-treatment. The induction of *OsIPT4* and *OsIPT5* by nitrate was completely inhibited by the MSX pre-treatment, although *NR* induction was not affected (Fig. 6B). This implied that nitrate-specific regulation of *OsIPT* gene induction is negligible or very minor under the given conditions.

**Fig. 7:**
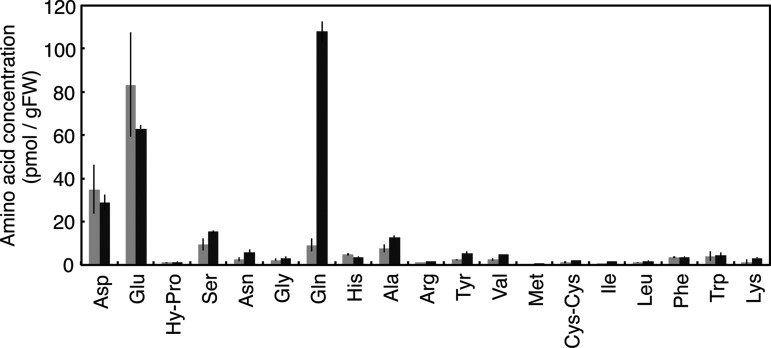
Changes in the concentrations of free amino acids in rice roots after application of ammonium. Rice seedlings were hydroponically grown in tap water for 11 d after sowing and transferred to nitrogen-free culture medium for 3 d. Then, the roots were dipped into the culture medium containing 1 mM KCl (light gray) or 1 mM NH_4_Cl (dark gray) for 30 min. Roots were harvested in triplicate, and the amino acid contents were analyzed. Bars represent mean values with the SD. The detailed data are provided in Supplementary Table S3.

Next, we analyzed the responses of amino acid concentrations in rice roots to inorganic nitrogen sources. Rice seedlings were grown for 2 weeks after germination without a nitrogen source, before ammonium or nitrate were supplemented. Upon exposure to ammonium, glutamine accumulated drastically in the roots within 30 min, and increased further over 2 h (Fig. 7; Supplementary Table S3). On the other hand, concentrations of aspartic and glutamic acids were decreased, and other amino acids showed no significant effects. In the nitrate treatment, a small but increased concentration of glutamine was also observed (Supplementary Table S3). These results were consistent with the positive effects of exogenously applied glutamine on the induction of *OsIPT4* and *OsIPT5* expression.

### Characterization of *OsIPT4* knock-down transgenic rice

To evaluate the contribution of *OsIPT4* to nitrogen-dependent cytokinin accumulation, *OsIPT4*-repressed transgenic rice lines were generated through RNA interference (RNAi) techniques. We made two types of construct in which the targets of RNAi were designed to be in the 5′- and 3′-untranslated regions of *OsIPT4* (Supplementary Fig. S4A). Two independent T_1_ transgenic lines in which the *OsIPT4* expression levels were reduced were selected for each construct. In these RNAi lines, basal expression levels of *OsIPT4* were reduced to about 50% and there was no induction in response to ammonium (Supplementary Fig. S4B). As for other *OsIPT* genes, the expression of *OsIPT1*, *OsIPT2* and *OsIPT3* was reduced in the roots of these RNAi lines (Supplementary Fig. S5). However, the basal expression levels of these three *IPT* genes in roots were very low (Fig. 3) and not affected by ammonium (Supplementary Fig. S5). Thus, the decrease in *OsIPT4* expression was the dominant effect in these RNAi lines, and we evaluated *OsIPT4* function in these lines.

**Fig. 8:**
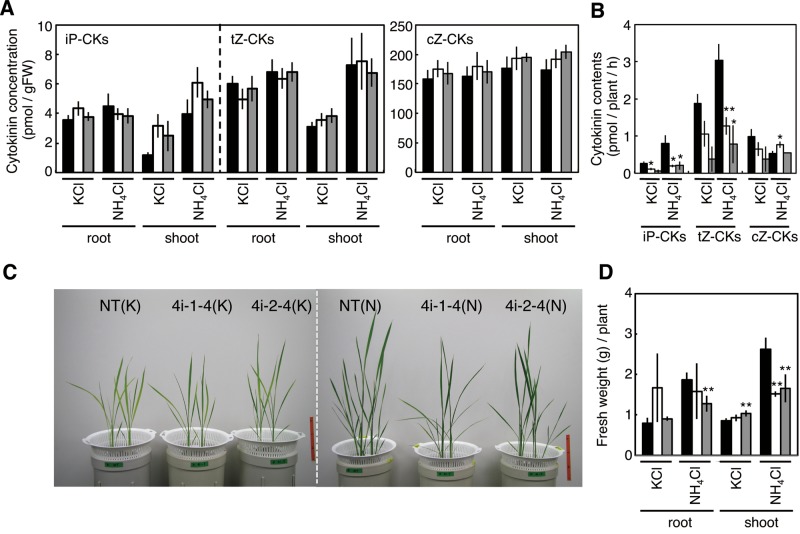
Characterization of *OsIPT4*-repressed transgenic lines. (A) Comparison of cytokinin concentrations. Non-transformant (black), 4i-1-4 (white) and 4i-2-4 (light gray) seedlings were hydroponically grown in tap water for 2 weeks and then transferred to culture medium containing 1 mM NH_4_Cl or 1 mM KCl. After 6 h, roots and shoots were harvested and cytokinin contents were quantified. (B) Comparison of cytokinin amounts exuded from the xylem. Asterisks indicate significant differences between non-transformants and the *OsIPT4*-repressed lines as established by Student’s *t*-test (**P < 0.01; *P < 0.05). (C and D) Comparison of growth. Rice plants were first grown in pH-controlled water for 2 weeks and then in liquid culture medium containing 1 mM NH_4_Cl (N) or 1 mM KCl (K) for 3 weeks. (C) Plants at 5 weeks after sowing. (D) Quantification of root and shoot fresh weight. Mean values ± SD from four plants of each treatment are shown. NT, non-transformant. Asterisks indicate significant differences between non-transformants and *OsIPT4*-repressed lines, according to Student’s *t*-test (**P < 0.01).

To examine whether *OsIPT4* induction contributes to the nitrogen-responsive cytokinin accumulation, we established the T_3_ RNAi lines and monitored cytokinin concentrations following ammonium application. Contrary to our expectation, no significant differences were found between non-transformants and the RNAi lines at the whole-organ level (Fig. 8A). Since *OsIPT4* is expressed in the vasculature (Fig. 4), and because major ammonium-induced changes occurred in the xylem-mobile nucleosides including iPR and tZR (Fig. 1; Supplementary Tables S1, S2), we analyzed cytokinin contents in stem base exudates which are derived mainly from xylem sap. In non-transformant control plants, the contents of tZ- and iP-type cytokinins in the exudate were increased following ammonium exposure (Fig. 8B). However, in both RNAi lines, the cytokinin contents in KCl-treated controls were decreased compared with non-transformants, and ammonium was ineffective. These results suggested that a reduction of the nitrogen-dependent induction of *OsIPT4* expression affects cytokinin export from roots via the xylem.

To study the effects of a deficiency of OsIPT4 function on growth, the T_3_ RNAi lines were hydroponically grown with 1 mM NH_4_Cl or KCl. The two independent *OsIPT4*-repressed lines showed retarded shoot growth compared with non-transformants in the presence of abundant nitrogen, whereas the differences in root growth were not significant (Fig. 8C, D). When the transgenic lines were grown for prolonged periods in a greenhouse, the retardation of shoot growth gradually declined and finally disappeared (data not shown). Thus, the effect of *OsIPT4* repression on shoot growth is most pronounced during early growth stages.

### Conservation of glutamine-dependent regulation of *IPT* in Arabidopsis

**Fig. 9:**
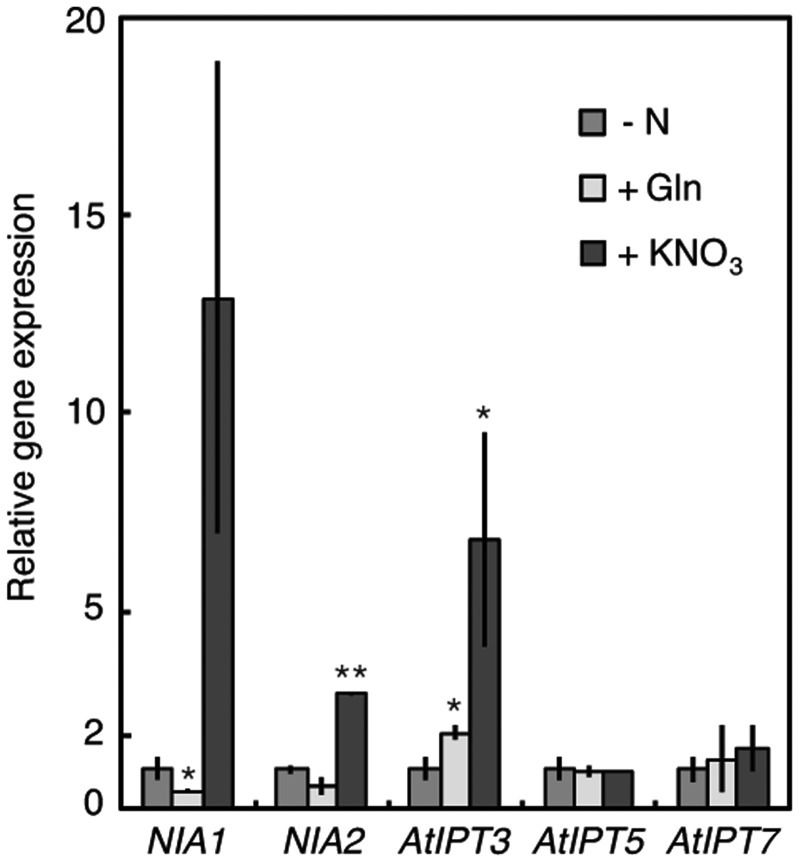
Effects of MSX pre-treatment on the induction of *AtIPT* transcript accumulation by exogenous nitrogen compounds. Arabidopsis seedlings were grown under standard conditions for 2 weeks after germination and transferred to nitrogen-free medium for 5 d. The seedlings were pre-treated with 1 mM MSX for 2 h and then incubated for 2 h with nitrogen-free medium (–N), 50 mM glutamine (+Gln) or 10 mM KNO_3_ (+KNO_3_) solution containing 1 mM MSX. Total RNA prepared from roots was subjected to qPCR which was performed in triplicate. The amounts of transcripts were normalized to that of the *Actin 2* transcript. The experiment was independently repeated twice. The values were normalized with respect to those obtained in the nitrogen-free medium, and mean values with the SD are shown. Asterisks indicate significant differences between the nitrogen-free treatment and the Gln or nitrate treatment, according to Student’s *t*-test (**P < 0.01; *P < 0.05).

To see whether the glutamine-related regulation is conserved in Arabidopsis *IPT* regulation, we examined the effects of MSX pre-treatment followed by nitrate and glutamine exposure in Arabidopsis (Fig. 9). *AtIPT3* expression in roots was induced by exogenous nitrate even after MSX pre-treatment. Glutamine application also increased the accumulation of *AtIPT3* transcripts about 2-fold (Fig. 9). Although this increase seemed small compared with the nitrate response, it consistently occurred in two independent experiments (Fig. 9). Thus, the glutamine-related regulatory mechanism of *IPT* expression exists in rice as well as in Arabidopsis, although the nitrate-specific regulation is the major one in Arabidopsis.

## Discussion

In previous studies on Arabidopsis, nitrogen-dependent de novo cytokinin biosynthesis was shown to be regulated in a nitrate-specific manner (Miyawaki et al. 2004, Takei et al. 2004). Here we demonstrated the involvement of glutamine metabolism in an additional regulatory system. We found that in rice, multiple *IPT* genes respond to exogenous inorganic nitrogen sources in a nitrogen-non-specific manner (Fig. 2), and that at least *OsIPT4* and *OsIPT5* respond to glutamine or a related compound as a metabolic signal (Fig. 6). In addition, the glutamine-related regulation of *IPT* expression appears to be functional also in Arabidopsis (Fig. 9). Since cytokinin activity was enhanced not only by nitrate but also by ammonium in barley and maize (Samuelson and Larsson 1993, Sakakibara et al. 1998, Sakakibara et al. 1999), the regulation of cytokinin biosynthesis by glutamine metabolism might be common in plants.

It is unlikely that the nitrate-specific regulation of cytokinin biosynthesis is restricted in Arabidopsis, because most plants preferentially utilize nitrate as the inorganic nitrogen source, and because a supply of nitrate to wheat (*T. aestivum* L.) that had been grown with ammonium further increased cytokinin levels (Garnica et al. 2010). At present, it is unclear whether the nitrate-specific regulation of *IPT* expression has been lost in rice. So far as we could determine, no data supporting the involvement of nitrate-specific regulatory steps in rice cytokinin metabolism are available. As ammonium is the major inorganic nitrogen source in paddy fields, the nitrate-specific regulation of cytokinin biosynthesis might have degenerated during domestication. Whatever the case, plants have evolved multiple modes of regulation of cytokinin de novo biosynthesis through nitrate, the basic nitrogen form taken up from the soil, and through glutamine metabolism, a key component of nitrogen assimilation. Thus the nitrogen-dependent control of cytokinin activity appears to be important for the modulation of plant growth and development.

At present, we do not know if glutamine itself functions as a genuine signal. The amido moiety of glutamine is utilized by glutamine-amidotransferase superfamily enzymes to form a wide variety of nitrogen-containing compounds (Massiere and Badet-Denisot 1998). Such metabolites might play a critical role in the control of *IPT* expression. We tried to evaluate the effects of 6-diazo-5-oxo-l-norleucine, a potent inhibitor of all glutamine-amidotransferases, in tests analogous to the MSX experiments reported above. However, the 6-diazo-5-oxo-l-norleucine pre-treatment itself severely affected the accumulation of transcripts, probably due to its cytotoxicity (data not shown), making it impossible to obtain unambiguous results. Glutamine is a major form of nitrogen in rice stem base sap (i.e. xylem sap) (Fukumorita and Chino 1982) and phloem sap (Hayashi and Chino 1990), and nitrogen-regulated IPTs are expressed in the vasculature (Fig. 4). Therefore, glutamine surrounding the translocation conduits could be an indicator of the internal nitrogen status for the regulation of cytokinin production. Exogenously supplied glutamine was demonstrated to affect the expression of a number of genes (Shiraishi et al. 1992, Vincentz et al. 1993, Watanabe et al. 1994, Hirose et al. 1997, Sonoda et al. 2003, Konishi and Yanagisawa 2010). While it remains unclear whether all of these genes are regulated by the same mechanism, it is intriguing that *NADH-GOGAT1* and *OsIPT4* apparently are. In sink organs, NADH-GOGAT1 plays an important role in the reutilization of nitrogen imported from source organs (Tabuchi et al. 2007, Tamura et al. 2010). In addition, GS1;2 and NADH-GOGAT1 are key elements in the primary assimilation of ammonium in roots (Tabuchi et al. 2007, Tamura et al. 2010, Funayama et al. 2013). GOGAT utilizes glutamine and supplies glutamate to GS to drive the GS/GOGAT cycle, an essential process in nitrogen assimilation (Miflin and Lea 1980). Nitrogen metabolic and developmental processes could be coordinated through a linkage between IPT regulation and GOGAT function.

In rice, cZ-type cytokinins are more abundant than iP- and tZ-type cytokinins (Kojima et al. 2009), but only the latter are produced in response to exogenous nitrogen (Fig. 1). In addition, levels of cZ-type cytokinins were not affected in *OsIPT4*-repressed lines (Fig. 8A, B). The biosynthesis pathway of cZ has not yet been elucidated in rice, but our results indicate that nitrogen-responsive IPTs are not directly involved in its de novo biosynthesis.

Our observations of GFP-tagged IPTs indicate that nitrogen-responsive IPTs are differentially localized in plastids and mitochondria in rice. Promoter::reporter analyses showed that *OsIPT4*, *OsIPT5* and *OsIPT8* were expressed in the phloem while *OsIPT7* was expressed in the xylem parenchyma in leaves (Fig. 4). Mitochondria are abundant in xylem parenchyma cells in rice leaves (Botha et al. 2008), which is in line with the expression of mitochondria-directed OsIPT7 in this tissue. On the other hand, GFP-tagged OsIPT4, OsIPT5 and OsIPT8 were localized in plastids (Fig. 5) and so was the Arabidopsis nitrogen-responsive IPT3. Given that *OsIPT4* and *OsIPT8* are the major *IPT* genes expressed in leaves and roots, it seems that the major route providing prenyl for nitrogen-dependent cytokinin de novo biosynthesis is the methylerythritol phosphate pathway as it is in Arabidopsis (Kasahara et al. 2004).

When we fused full-length OsIPT4, OsIPT5 and OsIPT8 to GFP, GFP fluorescence was observed on nucleoids in plastids (Fig. 5). Since we used a heterologous transient expression system and a strong promoter, we cannot exclude the possibility of an artificial localization. Further detailed studies will be needed to clarify this point.

Our analysis at the seedling stage suggested that the repression of *OsIPT4* affects cytokinin translocation in the xylem. The observed growth retardation could be attributed at least in part to the decreased cytokinin transfer. At the seedling stage, *OsIPT4* transcripts are much more abundant in roots than in shoots (Fig. 3). Consequently, root-borne cytokinin synthesized mainly by OsIPT4 might be important for normal shoot growth during early growth stages. In older plants, other IPTs might compensate for any malfunction of OsIPT4. On the other hand, we could not detect significant changes in cytokinin concentrations at the whole-organ level (Fig. 8). At present, we do not have an explanation for this discrepancy, but homeostatic regulation of cytokinin metabolism might mask local concentration differences at the whole-organ level.

In this study, we characterized the dual regulation system of cytokinin biosynthesis by nitrogen in rice, but we still do not understand how plants organize this system to optimize their growth and development under conditions of limiting and variable nitrogen availability. Further studies should focus on functional differentiations of the regulatory system. As for the growth optimization, other phytohormones probably interact with cytokinin-mediated growth regulation. For instance, recent studies indicated an interaction between plant architecture, macronutrients and phytohormones such as strigolactone (Yoneyama et al. 2007, Minakuchi et al. 2010, Umehara et al. 2010, Yamaguchi and Kyozuka 2010, Seto et al. 2012, Yoneyama et al. 2012). For a deeper understanding at the whole-plant level, we will have to study the nitrogen-dependent dual regulation system in the context of hormone–hormone interactions.

## Materials and Methods

### Plant materials and growth conditions

Rice (*O. sativa*) cultivar Nipponbare and *A. thaliana* ecotype Columbia were used in this study. For experiments on nitrogen responses, rice seeds were incubated in distilled water at 30°C for 2 d in the dark. The germinated seeds were sown on mesh trays floating on tap water adjusted to pH 5.5 using HCl and grown for 11 d in an environment-controlled greenhouse with a 12 h light (30°C)/12 h dark (25°C) photoperiod. Seedlings then were transferred to one-quarter-strength nutrient solution (Makino et al. 1983) without nitrogen source and further grown for 3 d. The media for treatment with 1 mM NH_4_Cl, 1 mM KNO_3_, 1 mM KCl or 1 mM MSX were prepared using the one-quarter-strength nutrient solution without nitrogen source. For characterization of *OsIPT4*-repressed lines, non-transformants and *OsIPT4*-repressed lines were grown in the growth chamber for a total of 5 weeks: in the first 2 weeks, plants were grown in pH-controlled water, then transferred to the liquid culture medium with 1 mM NH_4_Cl or 1 mM KCl for 3 weeks. The liquid culture medium was renewed every day to avoid nutrient depletion. For xylem sap collection, rice plants were first grown with normal liquid medium for 5 weeks and then transferred to nitrogen-free medium for 2 weeks to impose nitrogen starvation. Then, the plants were transferred to liquid medium containing 1 mM NH_4_Cl or 1 mM KCl. After 3 h, the shoot was cut off about 10 mm above the root/shoot transition with a razor blade, and root-pressure exudate was collected for 2 h. Samples were dried using a vacuum dryer, and subjected to cytokinin analysis.

Arabidopsis was grown on Molecular Genetics Research Laboratory (MGRL) growth medium (Fujiwara et al. 1992) in vertical agar plates containing 1% sucrose under 100 µmol m^−2 ^s^−1^ fluorescent light and long-day conditions (16 h light/8 h dark) at 22°C for 2 weeks. The seedlings were transferred to nitrogen-free MGRL-based vertical agar plates for 5 d, with CaCl_2_·2H_2_O and KCl instead of Ca(NO_3_)_2_·4H_2_O and KNO_3_ at equivalent quantities.

### Quantification of cytokinins

Extraction and determination of cytokinins from about 100 mg of fresh rice tissues were performed as described previously using ultra-performance liquid chromatography (UPLC)-tandem mass spectrometry (AQUITY UPLC™ System/XEVO-TQS; Waters) with an ODS column (AQUITY UPLC BEH C_18_, 1.7 µm, 2.1×100 mm, Waters) (Kojima et al. 2009).

### Quantitative PCR analysis

Total RNA was prepared from plant samples using the RNeasy Plant Mini Kit (Qiagen) according to the manufacturer’s instructions. Single-stranded cDNA was synthesized using the SuperScript® III First-Strand Synthesis System (Invitrogen) with oligo(dT)_12–18_ primers. Quantitative real-time PCR (qPCR) analysis was carried out with the StepOne Plus Real-Time PCR System (Applied Biosystem) using gene-specific primers (Supplementary Table S4) and the KAPA SYBR® FAST ABI Prism® 2X qPCR kit (Kapa Biosystems) according to the manufacturer’s protocol. To calculate the absolute quantity of each transcript, purified plasmids containing the target sequences of the primers were used to obtain linear standard curves.

### Histochemical analysis

Genomic DNA containing the putative promoter regions of *OsIPT4* (–3,051 to +296 bp from the first ATG codon)*, OsIPT5* (–2,722 to +257 bp), *OsIPT7* (–3,001 to +198 bp) and *OsIPT8* (–2,810 to +272 bp) were amplified by PCR with genomic DNA using gene-specific primers containing a specific restriction site (Supplementary Table S4). The PCR products were cloned into the pCAMBIA-GUS vector (Hirose et al. 2005) at the restriction site. The resulting constructs were named *OsIPT4pro:GUS*, *OsIPT5pro:GUS*, *OsIPT7pro:GUS* and *OsIPT8pro:GUS.* Transgenic rice plants were generated by the *Agrobacterium tumefaciens*-mediated method (Hiei et al. 1994) using strain EHA105. Histochemical analysis of GUS activity was performed by the method of Jefferson (1987), modified by Kosugi et al. (1990). Details of the staining procedure were described previously (Hirose et al. 2005). The sections were incubated at 37°C for between 10 min and several hours in GUS reaction buffer.

### 5′-RACE

Total RNA was prepared from leaf blades of mature rice using the RNeasy Plant Mini Kit, and 5′-RACE was performed using the GeneRacer™ Kit (Invitrogen) with *OsIPT8*-specific primers (Supplementary Table S4) according to the instruction manual.

### Analyses of GFP fusion proteins

The coding regions of *OsIPT1–OsIPT5*, *OsIPT7* and *OsIPT8* were fused to the N-terminus of the sGFP(S65T) sequence of the pGWB5 vector (Nakagawa et al. 2007), whose expression is controlled by the CaMV 35S promoter. The expressed proteins were named OsIPT1–GFP to OsIPT5–GFP, OsIPT7–GFP and OsIPT8–GFP, respectively. The full length (with stop codon) of the coding sequence of *OsIPT1–OsIPT3* was also fused to the C-terminus of the sGFP(S65T) sequence of the pGWB6 vector (Nakagawa et al. 2007). The expressed proteins were designated as GFP–OsIPT1 to GFP–OsIPT3, respectively. These constructs were introduced into Arabidopsis root cells or rosette leaf cells by bombardment (PDS-1000/He, Bio-Rad) with 1 µm gold particles as described in the supplier’s protocol. For co-introduction of multiple constructs, the N-terminal region of AtGGPS6 was fused to the N-terminus of DsRed2 (*pGGPS6-DsRed2*), and so was the full coding sequence of AtFSD3 (*pAtFSD3-DsRed2*); both constructs were under the control of the CaMV 35S promoter. After overnight incubation in the dark, transient expression was observed by confocal laser-scanning fluorescence microscopy (Zeiss LSM510 META, Carl Zeiss).

### Determination of free amino acids

Rice plants hydroponically grown for 14 d after germination as described above were transferred to one-quarter-strength nutrient solution with 1 mM NH_4_Cl or 1 mM KCl. After 30 min and 2 h, roots were harvested and rapidly frozen in liquid nitrogen. Three biological replicates were prepared. The frozen roots were powdered in liquid nitrogen and then homogenized in 10 vols. of 10 mM HCl with 0.2 mM methionine sulfone as an internal control. The homogenate was centrifuged and the supernatant was filtered through Ultrafree-MC filters (Millipore). Amino acid contents in the resulting filtrate were determined using Pico·Tag® (Waters) with an HPLC System (Waters Alliance 2695 HPLC system/2475) according to the instruction manual.

### Generation of OsIPT4-repressed lines

Target regions for RNAi were amplified by PCR from rice cDNAs with specific primers (Supplementary Table S4). The resulting PCR products were cloned into the pENTR/D-TOPO vector (Invitrogen) according to the instruction manual. The plasmids were used for LR reactions using the Gateway LR clonase Enzyme Mix (Invitrogen) and the pANDA vector (Miki and Shimamoto 2004). Transgenic rice plants were generated by the *A. tumefaciens*-mediated method using strain EHA105.

## Supplementary data

Supplementary data are available at PCP online.

## Funding

This work was supported by the Ministry of Education, Culture, Sports, Science and Technology, Japan [a Grant-in-Aid for Scientific Research on Innovative Areas (No. 21114005) and an NC-CARP project].

## Supplementary Material

Supplementary Data

## Abbreviations

CaMV: *Cauliflower mosaic virus*
cZ: *cis*-zeatin
cZR: cZ riboside
cZRP: cZR 5′-phosphate
FNR: ferredoxin-NADP^+^ oxidoreductase
GFP: green fluorescent protein
GS: glutamine synthetase
GUS: β-glucuronidase
iP: *N*^6^-(Δ^2^-isopentenyl)adenine
iPR: iP riboside
iPRP: iPR 5′-phosphate
IPT: adenosine phosphate-isopentenyltransferase
MSX: l-methionine sulfoximine
NADH-GOGAT1: NADH-dependent glutamate synthase 1
NR: nitrate reductase
qPCR: quantitative real-time PCR
5′-RACE: 5′-rapid amplification of cDNA ends
RNAi: RNA interference
tZ: *trans*-zeatin
tZR: tZ riboside
tZRP: tZR 5′-phosphate
