# Supplementary material for: Nitrogen-Dependent Regulation of De Novo Cytokinin Biosynthesis in Rice: The Role of Glutamine Metabolism as an Additional Signal
Source: Plant Cell Physiol. 2013 Oct 10;54(11):1881–93. doi: 10.1093/pcp/pct127 (PMC3814184; doi:10.1093/pcp/pct127)
Supplement: Supplementary Data [file supp_54_11_1881__index.html]

Nitrogen-dependent regulation of de novo cytokinin biosynthesis in rice: the role of glutamine metabolism as an additional signal — Nitrogen-Dependent Regulation of De Novo Cytokinin Biosynthesis in Rice: The Role of Glutamine Metabolism as an Additional Signal — Nitrogen-Dependent Regulation of De Novo Cytokinin Biosynthesis in Rice: The Role of Glutamine Metabolism as an Additional Signal — Supplementary Data 

# Nitrogen-Dependent Regulation of De Novo Cytokinin Biosynthesis in Rice: The Role of Glutamine Metabolism as an Additional Signal

## Supplementary Data

files

**Files in this Data Supplement:**

- Supplementary Data - pdf file
- Supplementary Data - pdf file
- Supplementary Data - pdf file
- Supplementary Data - pdf file
- Supplementary Data - pdf file
- Supplementary Data - pdf file
- Supplementary Data - pdf file
- Supplementary Data - pdf file
- Supplementary Data - pdf file
