# Supplementary material for: Nitrogen-Dependent Regulation of De Novo Cytokinin Biosynthesis in Rice: The Role of Glutamine Metabolism as an Additional Signal
Source: Plant Cell Physiol. 2013 Oct 10;54(11):1881–93. doi: 10.1093/pcp/pct127 (PMC3814184; doi:10.1093/pcp/pct127)
Supplement: Supplementary Data [file supp_pct127_pcp-2013-e-00282-File011.pdf]

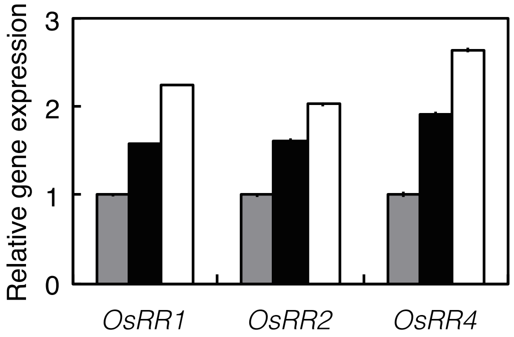

Supplementary Figure S1. Changes in the accumulation of *OsRR* transcripts in roots in response to nitrogen sources. Rice seedlings were hydroponically grown and treated with 1 mM NH_4Cl (black bars), 1 mM KNO_3 (white bars), or 1 mM KCl (gray bars) in the same manner as in Fig. 1, and roots were harvested after 2 h. Total RNA prepared from the samples was subjected to qPCR. The amounts of transcripts were normalized to the value found in the KCl treatment. qPCR was performed in triplicate, and mean values with SD are shown.
