# Supplementary material for: Nitrogen-Dependent Regulation of De Novo Cytokinin Biosynthesis in Rice: The Role of Glutamine Metabolism as an Additional Signal
Source: Plant Cell Physiol. 2013 Oct 10;54(11):1881–93. doi: 10.1093/pcp/pct127 (PMC3814184; doi:10.1093/pcp/pct127)
Supplement: Supplementary Data [file supp_pct127_pcp-2013-e-00282-File012.pdf]

```
reported  ------------------------------------------------------------
database  ------------------------------------------------------------
5' RACE   ----------------------------------------------TGGACTGAAG-GAG 13
genome    CTCCGTCTCCTTGCGCTGCGCGTTATATCGGCTCTGCCTATATAAGTGTGCTGAGGCGAC 120

reported  ------------------------------------------------------------
database  ------------------------------------------------------------
5' RACE   TAGA--------AGTGTTTTGGGTGGGCCGGCTTTATGAGCAGTCTCGGTTTGAAGATCC 65
genome    TGGGGCTCGGTGAGTGTTTTGGGTGGGCCGGCTTTATGAGCAGTCTCGGTTTGAAGATCC 180

reported  ------------------------------------------------------------
database  --------------------ATGGCGGCCGCGGCCGTCGCTGGCGTCGGAAGGGATGGTA 40
5' RACE   GCACCGTCGTCCGCTCACCTATGGCGGCCGCGGCCGTCGCTGGCGTCGGAAGGGATGGTA 125
genome    GCACCGTCGTCCGCTCACCTATGGCGGCCGCGGCCGTCGCTGGCGTCGGAAGGGATGGTA 240

reported  -----------------------------------------ATGGAGAGAAGCAGAGTCG 19
database  GCTTCGCCTCCCAGAAGCGGCCACGTCGGGTTAGTGTGAGAATGGAGAGAAGCAGAGTCG 100
5' RACE   GCTTCGCCTCCCAGAAGCGGCCACGTCGGGTTAGTGTGAGAATGGAGAGAAGCAGAGTCG 185
genome    GCTTCGCCTCCCAGAAGCGGCCACGTCGGGTTAGTGTGAGAATGGAGAGAAGCAGAGTCG 300
                                                   *******************

reported  GGGACGGTTGCTGCTGCTCCTGCTCTGGCCGCGGCGGGGTGGCGTCCACTACGGCGGTCC 79
database  GGGACGGTTGCTGCTGCTCCTGCTCTGGCCGCGGCGGGGTGGCGTCCACTACGGCGGTCC 160
5' RACE   GGGACGGTTGCTGCTGCTCCTGCTCTGGCCGCGGCGGGGTGGCGTCCACTACGGCGGTCC 245
genome    GGGACGGTTGCTGCTGCTCCTGCTCTGGCCGCGGCGGGGTGGCGTCCACTACGGCGGTCC 360
          ************************************************************
```

Supplementary Figure S2. Determination of the first ATG codon of the *OsIPT8* transcript by 5’ -RACE. Alignment of nucleotide sequences of predicted N-terminal regions of *OsIPT8*. The sequence reported by Sakamoto et al. (2006) is marked as ‘reported’ , the sequence registered in the Rice Genome Annotation Project (http://rice.plantbiology.msu.edu/) is marked as ‘database’ , the major sequence obtained by 5’ -RACE is marked as ‘5’ -RACE’ , and the sequence of the *OsIPT8* N-terminal region from the genomic sequence is marked as ‘genome’ . The first ATG codons in the three upper sequences are underlined in red. Asterisks indicate identical bases in the 4 sequences.
