# Supplementary material for: Nitrogen-Dependent Regulation of De Novo Cytokinin Biosynthesis in Rice: The Role of Glutamine Metabolism as an Additional Signal
Source: Plant Cell Physiol. 2013 Oct 10;54(11):1881–93. doi: 10.1093/pcp/pct127 (PMC3814184; doi:10.1093/pcp/pct127)
Supplement: Supplementary Data [file supp_pct127_pcp-2013-e-00282-File013.pdf]

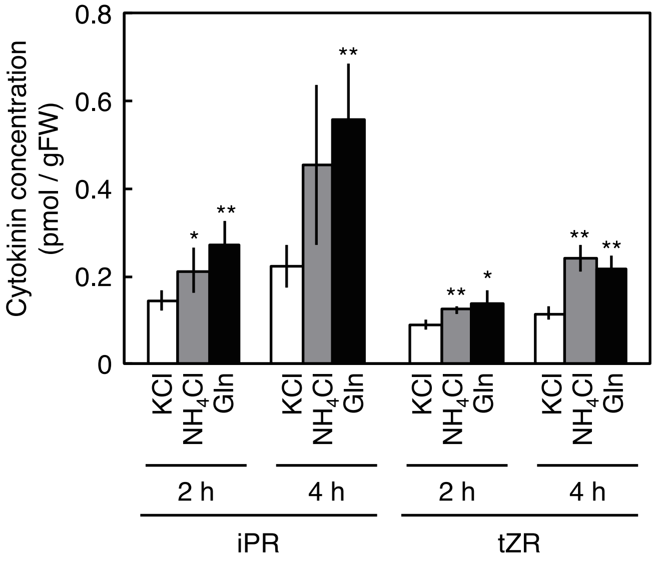

Supplementary Figure S3. Accumulation of cytokinins in rice roots in response to ammonium and glutamine. Rice seedlings were hydroponically grown in tap water for 11 days after sowing before transfer to nitrogen-free culture medium for 3 days. Then, the roots were dipped into culture media containing 1 mM NH_4Cl, 50 mM Glutamine, or 1 mM KCl. After the time indicated, roots were harvested in triplicate, and the cytokinin contents were quantified. Bars represent mean values with SD. *; $p < 0.05$, **; $p < 0.01$ (Student' s *t*-test, comparison with the KCl treatment).
