# Supplementary material for: Nitrogen-Dependent Regulation of De Novo Cytokinin Biosynthesis in Rice: The Role of Glutamine Metabolism as an Additional Signal
Source: Plant Cell Physiol. 2013 Oct 10;54(11):1881–93. doi: 10.1093/pcp/pct127 (PMC3814184; doi:10.1093/pcp/pct127)
Supplement: Supplementary Data [file supp_pct127_pcp-2013-e-00282-File014.pdf]

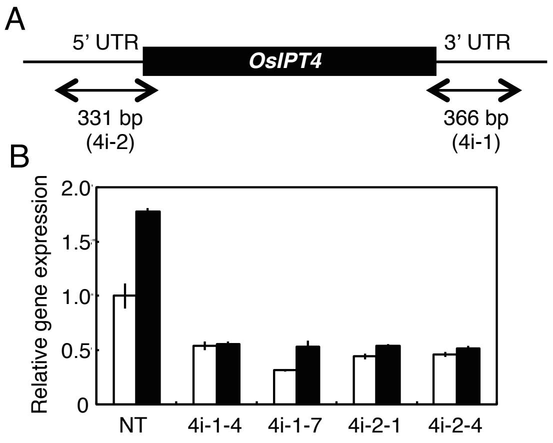

Supplementary Figure S4. Generation of two independent lines of *OsIPT4*-repressed transgenic rice by RNAi. (A) Schematic representation of RNAi constructs. The bar indicates the exon of *OsIPT4*, and the lines represent the 5' - and 3' -untranslated regions (UTR). Double-headed arrows indicate the region used for RNAi construction in the two independent lines, 4i-1 and 4i-2. (B) Effect of RNAi on the accumulation of the *OsIPT4* transcript. Rice seedlings were hydroponically grown and treated with 1 mM NH_4Cl (black bars) or 1 mM KCl (white bars) for 3 h in the same manner as in Fig. 1. Total RNA prepared from roots was subjected to qPCR. The amounts of transcripts were normalized to the value in non-transformant (NT) plants treated with KCl. qPCR was performed in triplicate, and mean values with SD are shown.
