# Supplementary material for: Nitrogen-Dependent Regulation of De Novo Cytokinin Biosynthesis in Rice: The Role of Glutamine Metabolism as an Additional Signal
Source: Plant Cell Physiol. 2013 Oct 10;54(11):1881–93. doi: 10.1093/pcp/pct127 (PMC3814184; doi:10.1093/pcp/pct127)
Supplement: Supplementary Data [file supp_pct127_pcp-2013-e-00282-File016.pdf]

Supplementary Table S1 Cytokinin concentrations in rice roots after exposure to a nitrogen source

		KCl				NH_4Cl				KNO_3			
**Time (h)**	**0**	**1**	**2**	**4**	**6**	**1**	**2**	**4**	**6**	**1**	**2**	**4**	**6**
						pmol g^{-1} fresh weight							
tZ	BQ	BQ	BQ	BQ	BQ	BQ	BQ	BQ	BQ	BQ	BQ	BQ	BQ
tZR	0.09	0.08	0.07	0.08	0.11	0.11	0.17	0.29	0.12	0.07	0.22	0.95	0.69
tZRPs	0.64	0.40	0.39	0.34	0.44	0.70	0.95	1.04	0.65	0.65	1.58	3.97	3.25
cZ	1.74	1.74	1.92	1.56	3.15	1.52	1.57	2.24	1.70	2.27	1.97	1.55	1.34
cZR	5.76	5.82	6.17	5.06	11.09	4.71	4.99	6.94	6.97	6.30	5.90	5.02	4.32
cZRPs	5.02	5.23	5.55	4.35	6.48	4.34	4.49	4.27	4.13	5.42	4.52	5.12	4.65
DZ	0.03	BQ	0.04	0.02	BQ	BQ	0.02	0.02	BQ	BQ	BQ	BQ	BQ
DZR	BQ	BQ	0.17	BQ	0.19	BQ	BQ	BQ	BQ	BQ	BQ	BQ	BQ
DZRPs	BQ	BQ	BQ	BQ	BQ	BQ	BQ	BQ	BQ	BQ	BQ	BQ	BQ
iP	0.49	0.35	0.44	0.31	0.35	0.30	0.39	0.27	0.36	0.23	0.24	0.29	0.30
iPR	0.61	0.33	0.21	0.33	0.37	0.27	0.40	0.51	0.46	0.28	0.24	0.46	0.34
iPRPs	6.32	4.87	3.69	3.65	3.83	4.72	4.87	4.46	4.27	5.26	4.92	7.67	6.61
tZ7G	BQ	BQ	BQ	BQ	BQ	BQ	BQ	BQ	BQ	BQ	BQ	BQ	BQ
tZ9G	BQ	BQ	BQ	BQ	BQ	BQ	BQ	BQ	BQ	BQ	BQ	BQ	BQ
tZOG	0.34	0.24	0.23	0.27	0.35	0.24	0.18	0.17	0.22	0.23	0.23	0.34	0.28
cZOG	296.0	287.4	283.5	301.7	299.6	272.8	279.2	273.7	291.4	285.9	293.7	286.0	281.3
tZROG	0.29	0.28	0.29	0.28	0.34	0.30	0.27	0.27	0.27	0.28	0.30	0.37	0.32
cZROG	130.0	122.7	124.7	130.3	124.8	121.9	128.9	124.3	127.3	123.2	125.0	119.4	116.0
tZRPsOG	0.05	0.06	0.05	0.04	0.05	0.05	0.04	0.05	0.07	0.04	0.05	0.05	0.05
cZRPsOG	28.36	29.77	29.43	27.32	24.17	27.23	24.40	30.00	32.20	26.43	33.03	21.78	25.40
DZ9G	BQ	BQ	BQ	BQ	BQ	BQ	BQ	BQ	BQ	BQ	BQ	BQ	BQ
iP7G	BQ	BQ	0.04	BQ	BQ	BQ	0.01	BQ	0.01	BQ	BQ	0.02	0.01
iP9G	1.52	1.71	1.57	1.56	1.41	1.43	1.50	1.44	1.42	1.45	1.54	1.56	1.41

Rice seedlings were hydroponically grown in tap water for 11 days after sowing and transferred to nitrogen-free culture medium for 3 days. Then, the roots were dipped into culture media containing 1 mM NH_4Cl, 1 mM KNO_3, or 1 mM KCl. After the time indicated, the roots were harvested in triplicate, and the cytokinin contents were quantified. Data are means of three experimental replicates. BQ, below quantification limit.

tZ, *trans*-zeatin; tZR, tZ riboside; tZRPs, tZR 5'-phosphates; cZ, *cis*-zeatin; cZR, cZ riboside; cZRPs, cZR 5'-phosphates; DZ, dihydrozeatin; DZR, DZ riboside; DZRPs DZR 5'-phosphates; iP, N^6-(Δ^2-isopentenyl)adenine; iPR, iP riboside; iPRPs, iPR 5'-phosphates; tZ7G, tZ-7-*N*-glucoside; tZ9G, tZ-9-*N*-glucoside; tZOG, tZ-*O*-glucoside; cZOG, cZ-*O*-glucoside; tZROG, tZR-*O*-glucoside; cZROG, cZR-*O*-glucoside; tZRPsOG, tZRPs-*O*-glucoside; cZRPsOG, cZRPs-*O*-glucoside; DZ9G, DZ-9-*N*-glucoside; iP7G, iP-7-*N*-glucoside; iP9G, iP-9-*N*-glucoside.
