# Supplementary material for: Nitrogen-Dependent Regulation of De Novo Cytokinin Biosynthesis in Rice: The Role of Glutamine Metabolism as an Additional Signal
Source: Plant Cell Physiol. 2013 Oct 10;54(11):1881–93. doi: 10.1093/pcp/pct127 (PMC3814184; doi:10.1093/pcp/pct127)
Supplement: Supplementary Data [file supp_pct127_pcp-2013-e-00282-File017.pdf]

Supplementary Table S2 Cytokinin concentrations in rice shoots after exposure to a nitrogen source

		KCl				NH_4Cl				KNO_3			
**Time (h)**	**0**	**1**	**2**	**4**	**6**	**1**	**2**	**4**	**6**	**1**	**2**	**4**	**6**
						pmol g⁻¹ fresh weight							
tZ	BQ	BQ	BQ	BQ	BQ	BQ	BQ	BQ	BQ	BQ	BQ	BQ	BQ
tZR	0.06	0.06	0.07	0.09	0.06	0.08	0.05	0.15	0.33	0.05	0.06	0.14	0.20
tZRPs	0.19	0.21	0.22	0.13	0.17	0.17	0.19	0.51	0.88	0.18	0.16	0.38	0.72
cZ	2.09	1.96	1.78	1.66	1.73	1.81	1.90	1.44	1.78	2.16	1.23	1.29	1.52
cZR	4.02	3.08	3.33	2.98	3.19	2.65	2.41	1.42	1.42	3.50	2.21	1.56	1.65
cZRPs	1.04	1.02	0.99	0.89	1.08	0.79	0.88	0.65	0.68	0.89	0.80	0.73	0.64
DZ	BQ	BQ	BQ	BQ	BQ	BQ	0.01	BQ	BQ	BQ	0.01	BQ	0.01
DZR	BQ	BQ	BQ	BQ	BQ	BQ	BQ	BQ	BQ	BQ	BQ	BQ	BQ
DZRPs	BQ	BQ	BQ	BQ	BQ	BQ	BQ	BQ	BQ	BQ	BQ	BQ	BQ
iP	0.34	0.28	0.42	0.54	0.41	0.49	0.74	0.88	1.26	0.54	0.42	1.21	0.52
iPR	0.03	0.03	0.07	0.08	0.06	0.06	0.30	0.16	0.29	0.07	0.03	0.09	0.07
iPRPs	0.71	0.61	0.58	0.87	0.75	0.95	1.13	2.58	4.49	0.94	0.95	1.27	1.39
tZ7G	BQ	BQ	BQ	BQ	BQ	BQ	BQ	BQ	BQ	BQ	BQ	0.20	0.26
tZ9G	BQ	BQ	BQ	BQ	BQ	BQ	BQ	BQ	BQ	BQ	BQ	BQ	BQ
tZOG	0.54	0.59	0.56	0.51	0.48	0.49	0.48	0.52	0.54	0.41	0.56	0.44	0.58
cZOG	319.6	334.7	320.1	338.2	314.6	303.8	306.4	312.1	310.2	317.7	322.3	328.1	333.3
tZROG	0.08	0.08	0.08	0.07	0.07	0.07	0.07	0.09	0.07	0.06	0.09	0.07	0.06
cZROG	36.98	41.83	41.15	40.32	36.43	37.74	38.90	36.56	32.19	36.48	42.60	35.95	36.84
tZRPsOG	0.03	0.03	BQ	BQ	0.02	BQ	BQ	BQ	BQ	0.02	BQ	0.02	BQ
cZRPsOG	9.37	9.94	8.30	9.00	8.20	7.59	6.87	8.44	5.61	9.86	7.54	7.61	7.41
DZ9G	BQ	BQ	BQ	BQ	BQ	BQ	BQ	BQ	BQ	BQ	BQ	BQ	BQ
iP7G	0.01	0.01	0.05	0.05	0.06	0.04	0.04	0.02	0.04	0.03	0.02	0.02	0.02
iP9G	1.70	1.59	1.32	1.81	1.92	2.26	1.79	1.99	1.62	1.72	1.86	1.87	1.80

Rice seedlings were hydroponically grown in tap water for 11 days after sowing and transferred to nitrogen-free culture medium for 3 days. Then, the roots were dipped into culture media containing 1 mM NH_4Cl, 1 mM KNO_3, or 1 mM KCl. After the time indicated, the shoots were harvested in triplicate, and the cytokinin contents were quantified. Data are means of three experimental replicates. BQ, below quantification limit.
tZ, *trans*-zeatin; tZR, tZ riboside; tZRPs, tZR 5'-phosphates; cZ, *cis*-zeatin; cZR, cZ riboside; cZRPs, cZR 5'-phosphates; DZ, dihydrozeatin; DZR, DZ riboside; DZRPs DZR 5'-phosphates; iP, N^6-(Δ^2-isopentenyl)adenine; iPR, iP riboside; iPRPs, iPR 5'-phosphates; tZ7G, tZ-7-*N*-glucoside; tZ9G, tZ-9-*N*-glucoside; tZOG, tZ-*O*-glucoside; cZOG, cZ-*O*-glucoside; tZROG, tZR-*O*-glucoside; cZROG, cZR-*O*-glucoside; tZRPsOG, tZRPs-*O*-glucoside; cZRPsOG, cZRPs-*O*-glucoside; DZ9G, DZ-9-*N*-glucoside; iP7G, iP-7-*N*-glucoside; iP9G, iP-9-*N*-glucoside.
