# Supplementary material for: Nitrogen-Dependent Regulation of De Novo Cytokinin Biosynthesis in Rice: The Role of Glutamine Metabolism as an Additional Signal
Source: Plant Cell Physiol. 2013 Oct 10;54(11):1881–93. doi: 10.1093/pcp/pct127 (PMC3814184; doi:10.1093/pcp/pct127)
Supplement: Supplementary Data [file supp_pct127_pcp-2013-e-00282-File018.pdf]

Supplementary Table S3 Concentrations of amino acids in rice roots after exposure to ammonium

	30 min			2 h		
	**KCl**	**NH_4Cl**	**KNO_3**	**KCl**	**NH_4Cl**	**KNO_3**
			pmol g^{-1} fresh weight			
Asp	35.0 ± 11.4	28.8 ± 3.6	52.4 ± 6.6	64.4 ± 31.3	35.8 ± 16.9	59.9 ± 14.5
Glu	83.2 ± 24.2	62.9 ± 1.7	122.3 ± 29.2	152.3 ± 51.0	77.8 ± 25.6	152.7 ± 11.0
Hy-Pro	1.1 ± 0.3	1.4 ± 0.3	BQ	5.2 ± 0.2	BQ	4.8 ± 1.1
Ser	9.4 ± 2.7	15.4 ± 0.6	14.0 ± 3.5	23.5 ± 15.0	20.2 ± 9.1	23.6 ± 7.1
Asn	2.7 ± 0.9	5.8 ± 1.2	4.6 ± 1.1	10.4 ± 11.5	7.7 ± 6.9	8.8 ± 7.8
Gly	2.3 ± 0.5	3.1 ± 1.1	BQ	15.0 ± 16.9	7.5 ± 1.9	9.0 ± 3.7
Gln	9.2 ± 2.9	107.8 ± 4.7**	16.8 ± 6.7	16.3 ± 7.6	162.1 ± 10.4**	25.5 ± 8.8
His	4.8 ± 0.3	3.3 ± 0.8	6.8 ± 1.4	13.7 ± 12.5	5.3 ± 1.7	8.5 ± 2.8
Ala	7.6 ± 1.9	12.7 ± 1.1	13.1 ± 4.0	17.5 ± 6.3	27.9 ± 24.3	17.6 ± 1.1
Arg	1.3 ± 0.7	1.6 ± 0.0	BQ	3.0 ± 1.3	2.2 ± 3.1	3.6 ± 0.2
Tyr	2.4 ± 0.4	5.3 ± 0.9*	4.1 ± 1.5	4.7 ± 1.3	7.9 ± 1.8	11.2 ± 8.3
Val	2.5 ± 0.6	4.8 ± 0.2*	3.9 ± 1.6	6.4 ± 2.3	15.4 ± 8.5	8.2 ± 1.9
Met	2.1 ± 0.7	0.7 ± 0.4	BQ	5.7 ± 5.6	3.7 ± 0.8	3.8 ± 1.2
Cys-Cys	1.3 ± 0.4	2.0 ± 0.1	1.9 ± 0.8	2.5 ± 1.6	2.3 ± 0.7	2.6 ± 0.8
Ile	0.8 ± 0.0	1.5 ± 0.1	0.9 ± 0.6	0.4 ± 0.6	4.8 ± 2.3	5.5 ± 1.8*
Leu	1.1 ± 0.1	1.8 ± 0.1	1.4 ± 0.5	2.1 ± 3.7	6.9 ± 4.1	11.4 ± 2.9
Phe	3.5 ± 0.3	3.3 ± 0.9	BQ	1.0 ± 1.8	4.8 ± 0.8	5.7 ± 0.5
Trp	4.0 ± 2.2	4.4 ± 1.6	4.2 ± 0.9	0.7 ± 1.2	5.5 ± 3.4	3.4 ± 0.4
Lys	1.2 ± 1.6	3.3 ± 0.1	3.3 ± 0.1	11.7 ± 7.2	8.6 ± 3.6	8.6 ± 2.6

Rice seedlings were hydroponically grown in tap water for 11 days after sowing and transferred to nitrogen-free culture medium for 3 days. Then, the roots were dipped into culture media containing 1 mM KCl, 1 mM NH_4Cl, or 1 mM KNO_3. After the indicated time, roots were harvested for determination of amino acid contents. Data are means ± SD of three biological replicates. Asterisks indicate significant differences between the value of the control treatment (KCl) and a nitrogen treatment (NH_4Cl or KNO_3) according to Student's *t*-test (*, $p < 0.01$, ** $p < 0.001$). BQ, below quantification limit.
