# Supplementary material for: Nitrogen-Dependent Regulation of De Novo Cytokinin Biosynthesis in Rice: The Role of Glutamine Metabolism as an Additional Signal
Source: Plant Cell Physiol. 2013 Oct 10;54(11):1881–93. doi: 10.1093/pcp/pct127 (PMC3814184; doi:10.1093/pcp/pct127)
Supplement: Supplementary Data [file supp_pct127_pcp-2013-e-00282-File019.pdf]

Supplementary Table S4 Primers used in this study

Primer name	Sequence (5' to 3')	Purpose for use	Underlined sequence
OsIPT1-F	ACCAAGCCCAAGGTTATCTTCGTGC	qPCR analysis for *OsIPT1*	
OsIPT1-R	TCGTCGGTGACCTTGTTGGTGATGA	qPCR analysis for *OsIPT1*	
OsIPT2-F	AAGTCCAAGCTCGCCATCTC	qPCR analysis for *OsIPT2*	
OsIPT2-R	GGTGACCTTGTTCGTGATGAT	qPCR analysis for *OsIPT2*	
OsIPT3-F	AGGCGAACACGTGGAGTCTG	qPCR analysis for *OsIPT3*	
OsIPT3-R	CCACCTTCAACTCCAGCACTCT	qPCR analysis for *OsIPT3*	
OsIPT4-F	GTACGAGTGCTGCTTCCTCTG	qPCR analysis for *OsIPT4*	
OsIPT4-R	CCAGATGCCCCTGGAGTAGT	qPCR analysis for *OsIPT4*	
OsIPT5-F	CAGCGTCAGCAGGAGCAT	qPCR analysis for *OsIPT5*	
OsIPT5-R	CGCGGCCGTGAACTCC	qPCR analysis for *OsIPT5*	
OsIPT7-F	AGGATACGAGGATGGTGGTGAT	qPCR analysis for *OsIPT7*	
OsIPT7-R	CCGTCATAGAGCTGAATCTTGTC	qPCR analysis for *OsIPT7*	
OsIPT8-F	CGAGGAGCTCGAGGAATACTT	qPCR analysis for *OsIPT8*	
OsIPT8-R	GTTGGCCTTGATCTCGTCTATC	qPCR analysis for *OsIPT8*	
FNR-F	GCAGGGAGCAGAAAAACAAG	qPCR analysis for *FNR*	
FNR-R	TCCCCTTCAAACCACAGAAG	qPCR analysis for *FNR*	
NR-F	CAAGTACGGCAAGCACTGGT	qPCR analysis for *NR*	
NR-R	ACGGAGTTGTCGGAGCTGTA	qPCR analysis for *NR*	
NADH-GOGAT-F	GTGCAGCCTGTTGCAGCATAAA	qPCR analysis for *NADH-GOGAT1*	
NADH-GOGAT-R	CGGCATTTCACCATGCAAATC	qPCR analysis for *NADH-GOGAT1*	
OsAct1REALf1	CGAGGCGCAGTCCAAGAG	qPCR analysis for *Actin*	
OsAct1REALre1	CCCAGTTGCTGACGATACCA	qPCR analysis for *Actin*	
AtNIA1F	CGCCATTATCCCACCATGA	qPCR analysis for *AtNIA1*	
AtNIA1R	GGAGAAGGAACGAGAGGAGGTT	qPCR analysis for *AtNIA1*	
AtNIA2F	ACGGCGTGGTTCGTTCTTAC	qPCR analysis for *AtNIA2*	
AtNIA2R	GGTTCTGGTGCGCCTTAGG	qPCR analysis for *AtNIA2*	
AtIPT3F	CATGGCGAATCTCTCCATTGA	qPCR analysis for *AtIPT3*	
AtIPT3R	AGTTGGAACCTCCAACGATGA	qPCR analysis for *AtIPT3*	
AtIPT5F	AGGATTTTCAGCGTGAAGCAA	qPCR analysis for *AtIPT5*	
AtIPT5R	CTATGATCGGGACACGGTCTCT	qPCR analysis for *AtIPT5*	
AtIPT7F	AACCTAACGGCCACCCAGTA	qPCR analysis for *AtIPT7*	
AtIPT7R	TGTTGTTCGCTGAGAGTTTCGA	qPCR analysis for *AtIPT7*	
ACT2-F	GTCCTCCTCACTTTCATCAGC	qPCR analysis for *Actin 2*	
ACT2-R	CATCAATTCGATCACTCAGAGC	qPCR analysis for *Actin 2*	
proIPT4-F	GCAAGCTTTCGGAGATTATTCCGATGAACC	Construction of *OsIPT4pro*:GUS	*Hin*dIII site
proIPT4-R	ATCCCGGGACCTTGTTCGTCACCACATCC	Construction of *OsIPT4pro*:GUS	*Sma*I site
proIPT5-F	ATCTCGAGAGCTTCCATGGTCGTCTCA	Construction of *OsIPT5pro*:GUS	*Xho*I site
proIPT5-R	ATCCCGGGAGCTTGTCGGCGTTGATCAC	Construction of *OsIPT5pro*:GUS	*Sma*I site
proIPT7-F	GCAAGCTTTGGTGAGCTGACCTGTCAGT	Construction of *OsIPT7pro*:GUS	*Hin*dIII site
proIPT7-R	ATGGATCCCTTGTCGGCGTTGACAACCTC	Construction of *OsIPT7pro*:GUS	*Bam*HI site
proIPT8-F	ATCTGCAGAACCTAACCATGTTGATATCCC	Construction of *OsIPT8pro*:GUS	*Pst*I site
proIPT8-R	ATCCCGGGTCGATGGAAAGCTTGGTCTT	Construction of *OsIPT8pro*:GUS	*Pst*I site
IPT8RACE	TCGAGGCCGTCGTACAGCTGAATCT	5' RACE specific primer for *OsIPT8*	
4i-1F	CACCAGCGTATTGGGAATTGAACG	Construction of *OsIPT4* RNAi line; 4i-1	
4i-1R	CTAGGTGTTCGACCGGTGTT	Construction of *OsIPT4* RNAi line; 4i-1	
4i-2F	CACCGCCGCTGCAATCTGAGAAG	Construction of *OsIPT4* RNAi line; 4i-2	
4i-2R	ACCCAAAATCAGAGGAGAAACC	Construction of *OsIPT4* RNAi line; 4i-2	
